# Effect of Thermal Aging on the Mechanical Properties of Rubber Composites Reinforced with Carbon Nanotubes

**DOI:** 10.3390/polym17070896

**Published:** 2025-03-27

**Authors:** Alžbeta Bakošová, Dana Bakošová, Petra Dubcová, Lukáš Klimek, Maroš Dedinský, Simona Lokšíková

**Affiliations:** 1 Department of Numerical Methods and Computational Modeling, Faculty of Industrial Technologies in Púchov, Alexander Dubček University of Trenčín, Ivana Krasku 491/30, 020 01 Púchov, Slovakia; petra.dubcova@tnuni.sk; 2 Department of Material Engineering, Faculty of Industrial Technologies in Púchov, Alexander Dubček University of Trenčín, Ivana Krasku 491/30, 020 01 Púchov, Slovakia; lukas.klimek@tnuni.sk; 3 Department of Material Technologies and Environment, Faculty of Industrial Technologies in Púchov, Alexander Dubček University of Trenčín, Ivana Krasku 491/30, 020 01 Púchov, Slovakia; maros.dedinsky@tnuni.sk (M.D.); simona.brigantova@tnuni.sk (S.L.)

**Keywords:** rubber nanocomposites, carbon nanotubes, natural rubber, mechanical properties, thermal aging

## Abstract

The incorporation of carbon nanotubes (CNTs) enhances the mechanical performance of rubber composites. This study examines the effect of single-walled CNTs (1–4 phr) on the properties of a natural rubber compound for sealing applications, both before and after thermal aging, as prolonged exposure to elevated temperatures can alter material properties, affecting durability and functionality. The researched nanocomposites were subjected to a series of mechanical tests, dynamic mechanical analysis, and surface investigation using atomic force microscopy (AFM). After adding 4 phr of CNTs, tensile and tear strength increased by 11.73% and 14.64%, respectively, while aging-related strength degradation was reduced by 5%. CNTs also increased complex modulus and hardness but reduced elongation at break and rebound resilience. Residual deformation in tensile and compressive set tests decreased. AFM analysis confirmed improved surface stability after thermal aging compared to the CNT-free compound.

## 1. Introduction

During thermal aging of a natural rubber (NR) compound, elevated temperatures cause oxidation, which impact the mechanical, physical, and chemical properties of the material. Oxygen reacts with the double bonds in polyisoprene molecules, which leads to the formation of peroxides, alcohols, ketones, and carboxylic acids. This causes the degradation of the polymer chains, which can result in loss of flexibility and strength. Thermal aging increases the material’s susceptibility to cracking. The hardness of the rubber material increases due to increased crosslink density, which restricts chain mobility. At elevated temperatures, some low-molecular components (e.g., antioxidants, plasticizers) can evaporate, which accelerates degradation. Vulcanizing agents, such as sulfur and accelerators, and their products can undergo changes during thermal aging, which can affect the strength of the bonds between the chains and reduce the resistance of the material. Thermal aging also causes optical and surface changes and the formation of microcracks on the surface [1,2].

To minimize the negative effects of thermal aging, stabilizers and antioxidants are added to rubber compounds to slow down the oxidation processes. The selection of appropriate fillers and vulcanization systems also plays an important role in increasing heat resistance. The rate of degradation depends on temperature, exposure time, the presence of antioxidants, and the composition of the rubber compound [3].

In the majority of applications, the NR is compounded with fillers to improve its material properties. In recent decades, rubber nanocomposites have been widely studied, as the addition of nanofillers, such as carbon nanotubes, graphene, nanoclays, and nano-oxides, to the elastomeric matrix can significantly its improve mechanical and thermal properties [4,5,6]. The carbon nanotubes exhibit exceptional properties including high tensile strength, thermal, and electrical conductivity; therefore, they are widely researched nanofillers [7,8].

To fully utilize potential of the CNTs, their uniform dispersion in the matrix is necessary. However, they tend to form agglomerates due to van der Waals forces between the CNTs, making them difficult to process. To improve their dispersion and surface interaction with the matrix, a variety of strategies have been devised, such as chemical and physical functionalization. Chemical functionalization involves the covalent attachment of functional groups to the outer walls of the CNTs. Direct covalent sidewall functionalization alters the hybridization from sp^2^ to sp^3^. Another chemical method is defect functionalization, which introduces covalent bonds at structural defects on the CNT sidewalls or open ends. These defects can be created through oxidative process, such as immersion in strong acids like H_2_SO_4_ or HNO_3_ [9,10,11]. Physical functionalization is a non-covalent method of surface modification by polymer wrapping or the absorption of various surfactants, which is less damaging to the CNT structure. The agglomeration can also be reduced mechanically, for example, by ultrasonication in suitable solvent, ball milling, or shear mixing [12,13,14].

There are numerous studies investigating the effect of the CNT–rubber composites and reporting improved mechanical properties, as well as thermal and electrical conductivity [15,16,17,18,19]. Many studies explore the use of CNTs in combination with other fillers, such as carbon black [20,21,22,23,24], silica [25], nanomaterials like graphene [26], and clay nanoparticles [13]. In the paper [26], the NR compound was reinforced with the CNTs and graphite particles, leading to an improvement in mechanical properties and thermal stability. In one study [27], the cure properties of NR/reclaimed rubber compound reinforced with single-walled carbon nanotubes (SWCNTs) were evaluated and the nanocomposite showed a decrease in minimum torque and increased scorch time and cure rate. The reinforcing effect of CNT on a NR/BR/IR/SBR compound was studied in the paper [28], where a rise in tensile strength, modulus, and hardness was observed, while elongation at break decreased. Another study [29] compared the performance of natural rubber (NR) composites modified with graphene and CNTs. While both fillers enhanced crosslinking density and reduced rolling resistance, Gr provided higher reinforcement in tensile strength, modulus, and hardness, but lowered manufacturing safety and cure rate. In contrast, CNTs improved cure rate, tear strength, tensile fatigue, and wear resistance, while reducing heat buildup and permanent compression set. In another study [30], the addition of silane-functionalized CNTs into styrene butadiene rubber process improved the crosslinking degree of vulcanizates. With the addition of 10 phr of functionalized CNTs-SiO_2_, a significant improvement in mechanical properties was observed. In the paper [31], multi-walled CNTs functionalized by a polydopamine coating (5 phr) were mixed with natural rubber (NR) via latex compounding, and the incorporation of functionalized CNTs increased tensile strength and thermal conductivity.

In this paper, the effect of adding single-walled CNTs on the mechanical properties of an NR compound for sealing applications filled with carbon black (30 phr) and silica (8 phr) is analyzed. The amount of the CNTs varied from 0 to 4 phr. The CNTs were functionalized to enhance their interaction with the NR matrix. This was achieved through oxidation in nitric acid (HNO_3_), followed by modification with nano SiO_2_-sol. While nitric acid treatment is a well-established method for CNT functionalization, its combination with nano-SiO_2_ sol in NR remains relatively unexplored.

The resulting nanocomposites were subjected to dynamic–mechanical analysis, tensile, hardness, tear strength, and rebound resilience tests. Compression and tensile sets were also determined, and their surface characteristics were examined using atomic force microscopy. The tested nanocomposites were subjected to thermal aging to evaluate the stability of their mechanical properties over time under elevated temperatures.

## 2. Materials and Methods

### 2.1. Materials

The rubber compounds with different proportions of single-walled carbon nanotubes (designated as NR-CNT 1 to NR-CNT 4) were produced. Commercial CNTs from Hongwu Material Technology Co., Ltd. (Guangzhou, China) were utilized. The used nanotubes had a diameter of 2 nm, a length of 5–20 μm, and a purity of 95%. The properties of these nanocomposites and the effect of temperature on their properties were compared with a compound without nanotubes (NR-0). The composition of the compounds is listed in Table 1, and the used ratios of the CNTs in Table 2.

**Figure 1:**
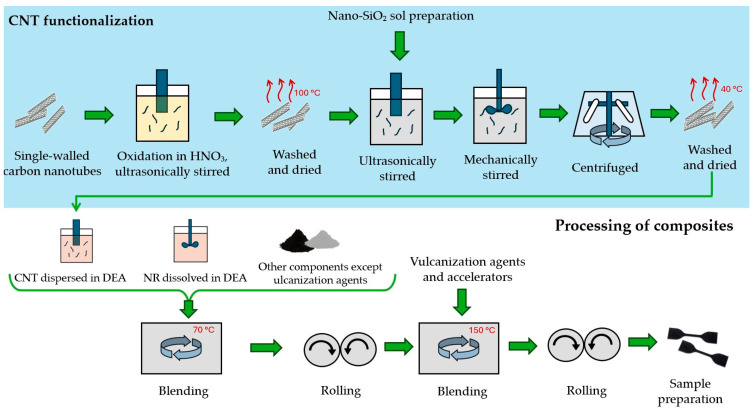
Scheme of nanocomposite production.

The production of the nanocomposites is schematically depicted in Figure 1. To ensure good dispersion in the rubber matrix, the CNT surface was functionalized in two steps. The first step involved functionalizing the CNTs with COOH groups to enhance their reactivity. The CNTs were oxidized and ultrasonically stirred in HNO_3_ (65%) at 120 °C for 24 h. Afterwards, they were washed with deionized water to remove excess nitric acid and dried in an oven at 100 °C for 5 h.

In the second step, the acid-treated CNTs were reacted with nano-SiO_2_ sol. Nano-SiO_2_ sol was prepared by mixing isopropyl alcohol, water, and HCl as a catalyst, then slowly adding tetraethoxysilane (98%) while stirring, in the ratio 4:1:0.05:4, respectively. The prepared nano-SiO_2_ sol was added at a ratio of 10% of the CNT volume to water with the ultrasonically dispersed oxidized CNTs. The mixture was sonicated for 5 h, then stirred at 50 °C for 2 h with a magnetic stirrer to enhance nano-SiO_2_ binding. Finally, it was centrifuged, washed, and dried at 40 °C for 12 h. This specific method was not previously published; however, a similar method was used in [30].

After functionalization, the CNTs were dispersed in ethanol and aromatic process oil (DAE). The mixture was then heated slightly above the boiling point of ethanol (80 °C) and further sonicated for 120 min using a mechanical ultrasonic sonicator to prevent agglomeration and achieve good nanotube dispersion.

Meanwhile, natural rubber was dissolved in ethanol with the addition of DAE. The solution was stirred at 80 °C for 120 min to ensure thorough mixing and the partial evaporation of ethanol.

The blending phase involved two mixing steps and evaporation of the excess solvent. The first step included mixing the rubber component with oil, carbon black, silica, resin, antioxidants, reclaimed rubber, stearin, and the functionalized CNTs in an oil solution. The blending was carried out using a Farrel Technolab BR 1600 Banbury mixer (Farrel Corporation One Farrel Boulevard, Ansonia, CT, USA). This process lasted 360 s at 70 °C, with a maximum mixing speed of 55 rpm and a pressure of 195 kPa. After mixing, the blend was rolled for 30 min on a Servitec double roller to enhance filler dispersion. In the second mixing step, in order to vulcanize the blend, the other components were added: insoluble sulfur, zinc oxide (ZnO), and CBS. The blend was processed for 150 s at 150 °C, then rolled again on a double roller to achieve the desired thickness for test sample preparation. Finally, the blend was left to cool and stabilize in a drying oven at laboratory temperature for seven days.

### 2.2. Methods

Transmission electron microscopy (TEM) and scanning electron microscopy (SEM) were utilized to obtain microscopic images of both the pristine CNTs and functionalized CNTs. To further evaluate CNTs after functionalization, Raman spectroscopy and Fourier-transform infrared spectroscopy (FTIR) were performed using a Nicolet iS50 spectrometer.

Thermal aging was performed in accordance with the standard ISO 188 [32], method A. The samples underwent thermal–oxidative aging in a circulation oven at 100 °C for 72, 96, 120, 144, and 168 h. Before further testing, the samples were left to relax for at least 24 h at 22 °C.

The following test were performed to evaluate changes after thermal aging, as well as the effect of the CNTs on material properties of the nanocomposites:

- The tensile tests in accordance with ISO 37 [33] were performed to determine the tensile strength, elongation, and tensile modulus. The working length of samples was 20 mm, thickness 2 mm, and width 6 mm. The loading speed was 100 mm·min^−1^. The test samples were manufactured in the shape of double-sided blades in accordance with the requirements of ISO 23529 [34]. For each material, five samples were tested before and after thermal aging.
- The hardness was measured using the Shore A method (ISO 7619-1 [35]). The tested samples were 6 mm thick and the measurement for each sample was performed five times.
- The tear strength was measured in accordance with the ISO 34-1 standard [36], method A. The test specimens were rectangular strips with dimensions of 15 mm, 100 mm, and thickness of 2 mm, with a 40 mm notch on one side of the specimen. The specimen was clamped in a tensile testing machine and stretched at a speed of 100 mm·min^−1^, enlarging the notch until the specimen ruptured.
- The rebound resilience test (ISO 4662 [37]) was carried out using the specimens with a circular cross-section (diameter of 44.6 ± 0.5 mm and thickness of 6.3 ± 0.3 mm). The measurement was performed using a Zwick 5109 testing machine.

To evaluate residual deformation after prolong exposure to a given strain load at different temperatures, tensile and compressive set tests were carried out. The tensile set tests were performed according to the STN 62 1452 standard [38]. The test specimens in the shape of double-sided blades (2 mm thick, 6 mm wide, and working length of 20 mm) were subjected to constant 25% and 50% elongation at the temperatures of 23 °C, 70 °C, and 100 °C. Specimens were deformed at a loading speed of 100 mm·min^−1^ until reaching the required deformation, which was maintained for 24 h. After a 30 min relaxation, the deformed length was measured. The tensile set was calculated according to formula:

(1) $$Tensile\;Set=\frac{L_{2}-L_{0}}{L_{1}-L_{0}}\;100\;(\%),$$

where *L*_0_ is length of unloaded specimen (mm), *L*_1_ is length of loaded specimen (mm), which corresponds to 25% or 50% elongation, and *L*_2_ is length of the specimen after relaxation (mm).

The compressive set tests (ISO 815 [39]) were performed on the type A test pieces, which were subjected to 25% compressive strain at temperatures of 23 °C, 70 °C, and 100 °C for 24 h, and residual deformation was measured after 30 min of relaxation.

Furthermore, dynamic–mechanical analysis (DMA) of rubber compounds was performed using a Pyris Diamond DMA analyzer (PerkinElmer, Waltham, MA, USA). The samples were prepared in the form of a strip with dimensions of 20 mm × 10 mm × 2.1 mm. They were subjected to tensile load in the temperature range −80 °C to 100 °C, using a heating rate of 5 °C·min^−1^, at a frequency of 1 Hz. A cryogenic nitrogen container was used to apply low temperatures. In addition to the temperature sweep, the frequency sweep was also carried out at frequencies of 0.01 Hz, 0.05 Hz, 0.2 Hz, 0.5 Hz, 1 Hz, 5 Hz, 10 Hz, 20 Hz, and 50 Hz, and a temperature of 20 °C.

Lastly, atomic force microscopy (AFM) (Microtestmachines Co., Ltd., Gomel, Belarus) was used to characterize surface properties. The AFM NT-206 microscope was utilized. The AFM allows for detailed investigation of the topography of materials down to the nanometer level, which is crucial for understanding the influence of the CNTs on the structure and properties of rubber compounds. An important aspect of testing is monitoring surface roughness before and after thermal aging. During thermal aging, the material degrades due to prolonged exposure to elevated temperatures, which can alter its mechanical properties and surface integrity. Measuring surface roughness before and after aging allows for the assessment of damage extent and material stability of the surface over time.

All experimental data, except microscopic images, were processed using MATLAB 2018a software.

## 3. Results and Discussion

### 3.1. Morphology of Functionalized Carbon Nanotubes

**Figure 2:**
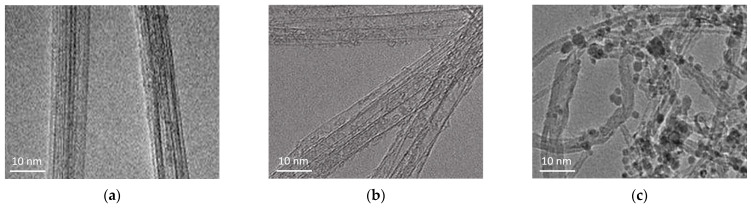
TEM images: (**a**) pristine CNTs; (**b**) CNTs oxidized in HNO_3_; (**c**) CNTs modified by nano-SiO_2_ after oxidation in HNO_3_.

Figure 2 presents the TEM images of pristine CNTs, CNTs oxidized in HNO_3_, and CNTs further modified with nano-SiO_2_ after oxidation. The pristine CNTs exhibit minimal defects or structural damage on their surface. After HNO_3_ treatment, small defects are visible on the nanotube surfaces due to chemical disruption of the walls and the introduction of oxygen functional groups (e.g., -COOH, -OH), which increase the CNTs’ reactivity. In the CNTs modified with nano-SiO_2_ sol, darker dot areas are visible, indicating the presence of silica nanoparticles, which may be chemically or physically bound to the CNTs.

**Figure 3:**
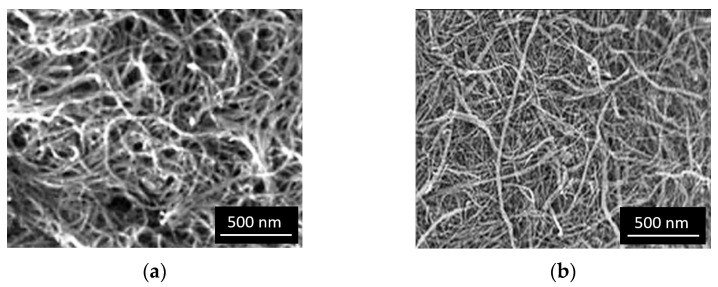
SEM images: (**a**) pristine CNTs; (**b**) CNTs modified by nano-SiO_2_ after oxidation in HNO_3_.

Excessive processing and mixing of the CNTs can lead to the breakage of the original micrometric nanotubes into smaller fragments. In the examined processed nanotubes, no drastic fragmentation of the CNTs was observed after chemical treatment, although a slight statistical reduction in CNT lengths cannot be completely ruled out. Analysis of SEM images (Figure 3) of treated CNTs allowed reliable measurements of the length of only a few nanotubes.

### 3.2. Fourier-Transform Infrared and Raman Spectroscopy

**Figure 4:**
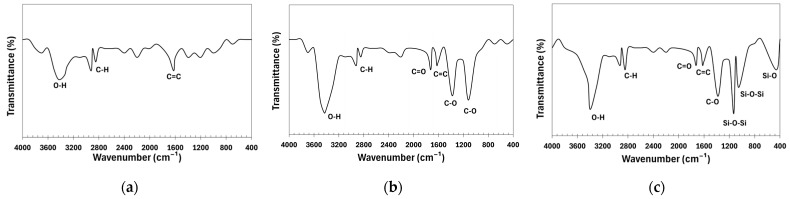
Fourier-transform infrared spectroscopy spectra: (**a**) pristine CNTs; (**b**) CNTs oxidized in HNO_3_; (**c**) CNTs modified by nano-SiO_2_ after oxidation in HNO_3_.

The FTIR spectra of the CNTs before and after functionalization are shown in Figure 4. The pristine CNT spectrum reveals key chemical features: the 1625 cm^−1^ band corresponds to C=C stretching, while bands at 2851 cm^−1^ and 2921 cm^−1^ indicate C-H stretching, suggesting organic impurities. The 3431 cm^−1^ band reflects hydroxyl (-OH) vibrations, likely due to adsorbed moisture or structural defects. Oxidation with HNO_3_ introduces new bonds, evident in the 1118 cm^−1^ band (C-O in -COOH, -C-O-C-, or -C-OH), 1380 cm^−1^ (bending vibrations of C-O in carboxyl group -COOH), 1626 cm^−1^ (C=C), and 1725 cm^−1^ (C=O in carboxyl and carbonyl groups). The 2849 cm^−1^ and 2920 cm^−1^ bands remain, likely from residual organic molecules. The 3433 cm^−1^ band indicates O-H stretching from hydroxyl groups. Treatment with nano-SiO_2_ introduces Si-O-related bands: 460 cm^−1^ (Si-O bending), 1052 cm^−1^, and 1132 cm^−1^ (Si-O-Si stretching), with 1132 cm^−1^ confirming SiO_2_ deposition [40]. The 1384 cm^−1^ band suggests C-O in carboxyl or nitro groups. Carboxyl groups are confirmed by the 1726 cm^−1^ band (C=O), while 2848 cm^−1^ and 2925 cm^−1^ (C-H) indicate residual organic impurities. The broad 3431 cm^−1^ band reflects hydroxyl (-OH) stretching [41]. These spectral changes confirm successful CNT functionalization with nitric acid (-COOH at 1726 cm^−1^) and silica deposition (Si-O-Si at 1132 cm^−1^).

**Figure 5:**
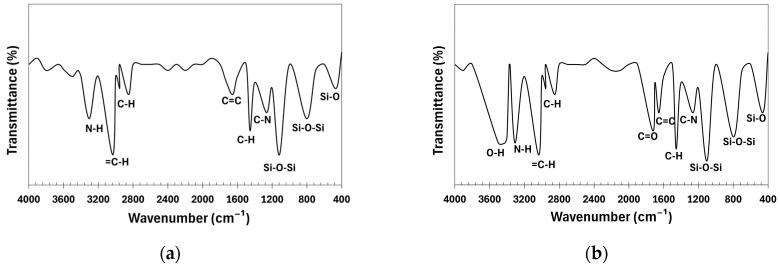
Fourier-transform infrared spectroscopy spectra of compounds: (**a**) NR-0; (**b**) NR-CNT 4.

Figure 5 presents the FTIR spectra of the NR-0 compound and NR-CNT 4 nanocomposite. In NR-0, characteristic absorption bands confirm the presence of key components: Si-O bending at 465 cm^−1^, Si-O-Si stretching at 1117 cm^−1^ and 804 cm^−1^ (ULTRASIL silica), and C-N stretching at 1265 cm^−1^ (6PPD, TMQ). Aromatic C=C stretching appears at 1654 cm^−1^ (TMQ), while C-H stretching at 2955 cm^−1^, 2853 cm^−1^, and 1455 cm^−1^ confirms natural rubber, reclaimed rubber, DAE, and stearin. The 3034 cm^−1^ band (=C-H stretching) and 3305 cm^−1^ (N-H stretching) indicate the presence of TMQ and 6PPD.

With functionalized CNTs, shifts and intensity changes occur: Si-O-Si stretching moves to 1102 cm^−1^ and 798 cm^−1^, and Si-O bending to 460 cm^−1^. The 1456 cm^−1^ (C-H deformation) and 1655 cm^−1^ (C=C stretching) bands intensify due to the addition of the functionalized CNTs. Hydrocarbon-related bands (2955 cm^−1^, 2852 cm^−1^, 3035 cm^−1^) remain unchanged, while N-H stretching at 3308 cm^−1^ increases due to hydroxyl groups on the CNTs. New functional groups (-COOH, C=O, -OH) appear, with absorption bands at 1724 cm^−1^ (C=O stretching) and 3423 cm^−1^ (O-H stretching), confirming CNT modification and interaction with the polymer matrix.

**Figure 6:**
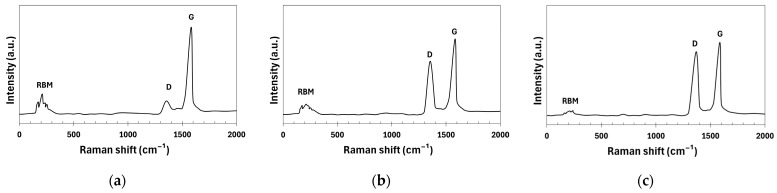
Raman spectra: (**a**) pristine CNTs; (**b**) CNTs oxidized in HNO_3_; (**c**) CNTs modified by nano-SiO_2_ after oxidation in HNO_3_.

Raman spectra are shown in Figure 6. In the high-frequency region of the spectrum of the pristine CNTs, two bands were observed: the G-band (1583 cm^−1^), which is related to the graphitic structure of the CNTs, and the weak D-band (1352 cm^−1^), which is associated with defects in the carbon structure. In the low-frequency part of the spectrum, there is a second region characteristic of the SWCNTs, known as the radial breathing mode (RBM). This mode describes the radial oscillations of carbon atoms, where the walls of the nanotube simultaneously expand and contract. This region is particularly prominent in CNTs with a diameter of less than 3 nm [42]. Oxidation in HNO_3_ causes structural disruption in CNTs, which is reflected in changes in the Raman spectrum. The D-band (1354 cm^−1^) showed increased intensity due to defects in the CNT structure, and the G-band (1584 cm^−1^) remained present but showed reduced intensity due to structural changes. The RBM band weakened because of the CNT disruption. Further modification with the nano-SiO_2_ sol, due to the coverage of the CNT with nanoparticles, led to a further increase in the intensity of the D-band (1369 cm^−1^) along with its slight shift, as well as additional weakening of the G-band (1586 cm^−1^) and an almost complete loss of the RBM band.

### 3.3. Test Results Before and After Aging

**Figure 7:**
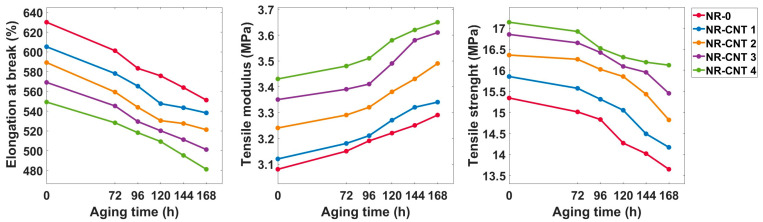
Tensile test results.

Tensile test results are presented in Table 3 and Figure 7. The incorporation of the CNTs increased both the strength and the tensile modulus of the nanocomposites. Prior to thermal aging, the NR-CNT 4 compound had 11.73% higher strength than the reference NR-0 compound. After thermal aging for 168 h at 100 °C, the strength of the NR-0 decreased by 11.02%, while NR-CNT 4 showed a reduction of only 5.95%.

The NR-CNT 4 nanocomposite also demonstrated the highest tensile modulus, which was 11.36% higher than that of the NR-0. Thermal aging led to an increase in the tensile modulus across all compounds, with the total percentage increase after 168 h ranging from 6.41% to 7.76%.

With an increasing amount of the CNTs, elongation at break decreased. Specifically, the NR-CNT 4 showed a 12.84% reduction in elongation at break compared to the NR-0. After thermal aging, the elongation at break declined. However, the rate of decrease did not significantly differ among the compounds with the varying CNT content. The total percentage decrease after aging ranged from 11.07% to 12.53%.

**Figure 8:**
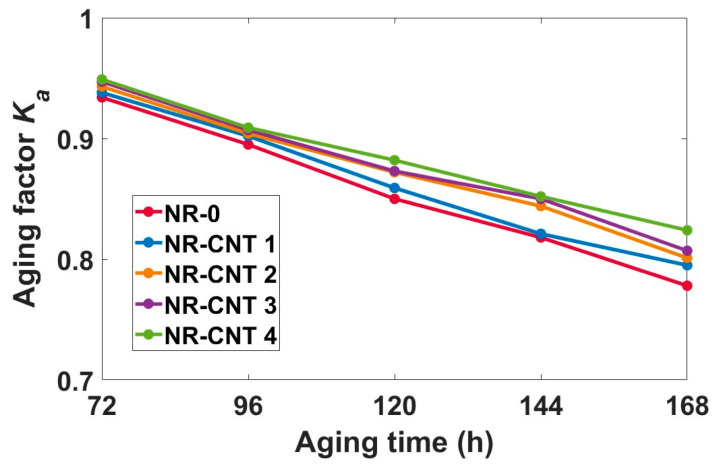
Values of aging factor after thermal aging.

From the tensile test results, the aging factor (*K_a_*) was determined to quantify change in mechanical properties, according to equation [43]:

(2) $$K_{a}=\frac{{TS}_{ag}E_{ag}\;}{{TS}_{ba}E_{ba}\;}\;\left(-\right),$$

where *TS_ba_* is tensile strength before aging (MPa), *E_ba_* is elongation at break before aging (%), *TS_ag_* is tensile strength after aging (MPa), *E_ag_* is elongation at break after aging (%). The product of strength and elongation is an indicator of the material’s ability to absorb mechanical energy during its deformation until rupture. The aging factor decreased with aging time. The values of *K_a_* are depicted in Figure 8. The addition of CNTs slowed the decline of *K_a_* values. Aging factor of the NR-0 decreased from 0.934 after 72 h of aging to 0.778 at 168 h of aging, while *K_a_* of the NR-CNT 4 decreased from 0.949 to 0.824.

**Figure 9:**
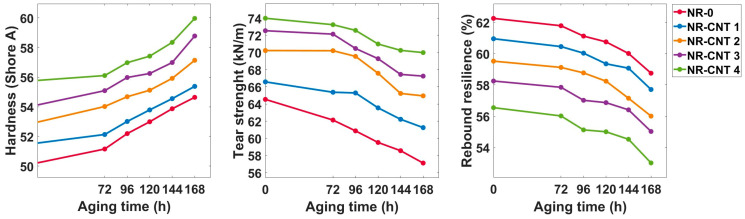
Hardness, tear strength, and rebound resilience test results.

The results of the hardness, tear strength, and rebound resilience measurements are depicted in Figure 9 and listed in Table 4. The hardness increased with an increasing CNTs content. Before thermal aging, the NR-CNT 4 had 11.09% higher hardness than the reference compound without the nanotubes. The thermal aging caused a rise in hardness values. Specifically, after aging for 168 h at 100 °C, the hardness of the NR-CNT 4 and NR-0 compounds increased by 8.82% and 7.51%, respectively.

The tear strength also increased with the CNTs content, and the addition of 4 phr of the CNTs resulted in 14.64% higher tear strength. The CNTs also contributed to reducing the rate at which tear strength declined during thermal aging. After 168 h of aging, the tear strength of the NR-0 decreased by 11.50%, while that of the NR-CNT 4 declined by only 5.41%.

Rebound resilience is a physical property of a material that describes its ability to recover its shape and energy after deformation caused by an impact load. The presence of the CNTs in the tested compounds caused a decrease in rebound resilience. A maximum decline of 9.16% was observed when comparing the unaged NR-CNT 4 and NR-0 compounds. After 168 h of thermal aging at 100 °C, the rebound resilience decreased within a range of 5.32–6.24%. Rubber compounds with lower rebound resilience are ideal for shock and vibration damping applications, such as seals and protective elements. These materials effectively absorb energy from the external environment and minimize vibration transmission to sensitive components.

During thermal aging, the formation of additional crosslinks restricts chain mobility, leading to a decline in elasticity. Prolonged exposure to elevated temperatures causes the material to harden, making the rubber stiffer. The primary factors contributing to strength reduction are chemical and physical degradation processes that compromise the polymer structure. In some cases, excessive crosslinking can occur, which diminishes the rubber compound’s ability to absorb mechanical stress, ultimately reducing strength. Additionally, certain additives that enhance strength and elasticity may degrade at high temperatures, further weakening the overall structure of the rubber.

The enhancement of polymer properties through the addition of the CNTs depends on the degree of crosslinking between polymer chains and the components of the composite. The high aspect ratio, large specific surface area, and good dispersion and alignment of the CNTs within the polymer matrix promote strong van der Waals interactions with polymer chains. These interactions contribute to the formation of nucleation sites for crosslinking agents, effectively initiating and supporting crosslinking reactions [44].

Additionally, functionalized CNTs can facilitate covalent bonding between polymer chains and crosslinking agents, thereby increasing crosslinking density in the composite. Chemically modified CNTs can directly participate in crosslinking reactions by interacting with polymer chains, leading to the formation of additional crosslinks.

The CNTs also exhibit excellent thermal conductivity. When well-dispersed in a polymer blend, they enhance heat transfer, promoting more uniform and efficient crosslinking reactions. Moreover, CNTs can influence the kinetics of crosslinking by altering reaction rates, activation energies, or reaction pathways, depending on the specific chemistry of the system [44,45,46].

The effects of CNTs depend on multiple factors, including the type of CNTs used, the polymer matrix, and the processing and manufacturing conditions of the composite.

### 3.4. Tensile and Compressive Sets Results

**Figure 10:**
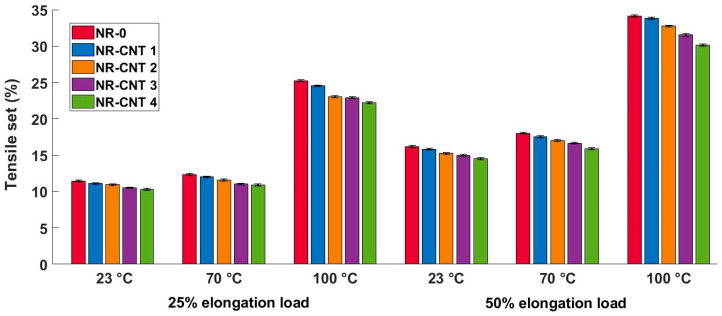
Tensile set test results at given temperature and elongation loads.

The tensile set test results are listed in Table 5 and shown in Figure 10. Tests performed at higher temperatures resulted in higher tensile set values. The addition of the CNTs decreased residual tensile deformation. The percentage difference in the tensile set values between the NR-CNT 4 and NR-0 compounds did not vary significantly across different test conditions. The tensile sets of the NR-CNT 4 were reduced by 10.14–11.88% compared to the NR-0.

**Figure 11:**
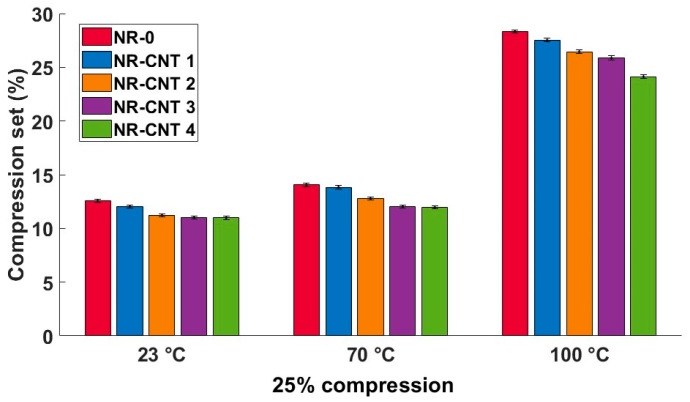
Compression set test results at given temperature, 25% compression strain.

The compression set test results are listed in Table 6 and shown in Figure 11. Similarly to the tensile set test results, the CNTs presence in the tested compounds caused a reduction in the residual deformation after relaxation at all tested temperatures. The compression set of the NR-CNT 4 was reduced by 12.64% at 23 °C, 14.95% at 70 °C, and 14.93% at 100 °C when compared to the NR-0. The residual deformation rose with an increasing temperature, at which the tests were performed. The reduction in the value of the compression set is particularly positive in applications such as seals.

The use of the CNTs in rubber compounds has a significant impact on the mechanical properties of polymer materials, including residual deformation after exposure to tensile or compressive strain load. The CNTs have a high elastic modulus and strength, which can increase the resistance of the rubber compound to deformation. With good dispersion, the CNTs help to form an effective network in the matrix, which ensures better stress transfer between polymer chains. This leads to a reduction in the concentration of local stress, which helps to prevent permanent changes in shape.

### 3.5. DMA Results

The DMA temperature and frequency sweeps were conducted. The tensile complex modulus, which provides a more comprehensive view of the material’s behavior, was determined. The storage modulus *E*′ describes not only material stiffness, but also the ability to absorb and release energy under dynamic conditions. The loss modulus *E*″ is a material parameter that expresses the viscous behavior of a material during the DMA. It indicates what part of the mechanical energy of the applied load is dissipated in the material due to internal friction and viscous processes [47].

**Figure 12:**
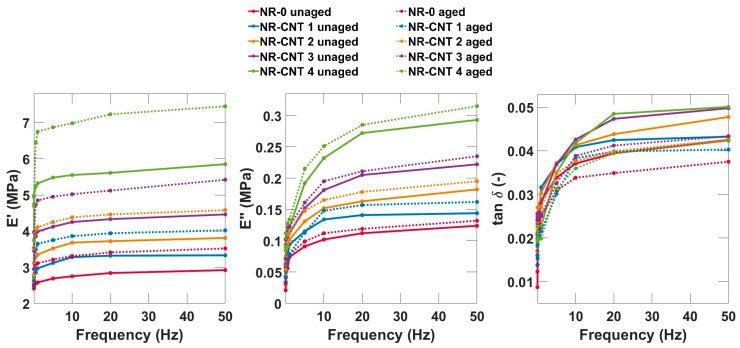
DMA frequency sweep (0.1–50 Hz) results at a temperature of 20 °C.

The results from the DMA frequency sweep are depicted in Figure 12. The elastic moduli *E*′, loss moduli *E*″, and *tan δ* for all compounds show increasing trends with increasing frequency. At low frequencies, the *E*′ values are lower because the molecules have enough time to reorient and relax, and the material has a lower ability to store mechanical energy effectively. At higher frequencies, the *E*′ value is higher because the molecule chains do not have time to rearrange, the deformation is mostly reversible, and the material has greater stiffness. Thermal aging, as well as addition of CNTs, caused an increase in both the elastic moduli *E*′ and loss moduli *E*″. The *tan δ* values slightly decreased.

**Figure 13:**
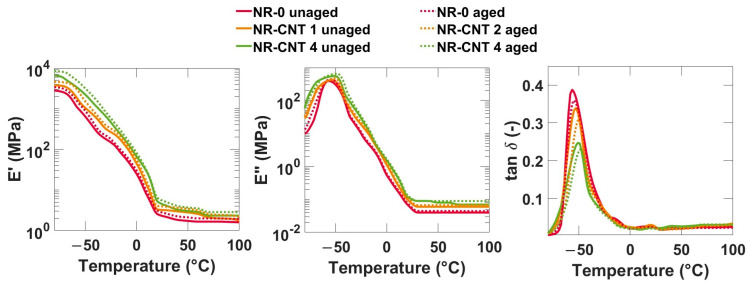
DMA temperature sweep (−80–50 °C) results at a frequency of 1 Hz.

The results from the DMA temperature sweep of representative compounds are shown in Figure 13. With rising temperatures, values of *E*′ decrease, and elastomers become softer and less stiff, as the molecules begin to have more kinetic energy, which leads to an increase in chain mobility. As the CNT content increased, the storage modulus *E*′ increased. The CNTs increased the stiffness of the investigated compounds. Thermal aging also caused an increase in the storage modulus. However, it is important to note that an increase in *E*′ can also negatively impact other material properties, such as rebound resilience, flexibility, and durability. This is especially true if the aging process is prolonged, or the material is exposed to fluctuating adverse conditions.

The values of the loss modulus *E*″ in the investigated compounds are significantly lower compared to the storage modulus *E*′, which means that the elastic properties prevail. The *E*″ increased slightly with the addition of nanotubes. Thermal aging caused a slight increase in the viscous modulus.

The tangent of phase angle *tan δ*, which is a ratio between the *E*″ and *E*′, expresses the phase shift between the applied stress and the strain response. It represents the ratio of dissipated loss energy to energy stored during the deformation cycle. The higher values of *tan δ* mean higher losses, which means a greater ability of the material to convert mechanical energy into heat. This leads to good damping properties, but also to higher heating of the material. Conversely, lower values represent lower losses, which is advantageous in applications where low hysteresis and high energy efficiency are desired, such as tires with low rolling resistance [48].

The glass transition temperature *T_g_* is determined from maximum of a temperature dependency of the *tan δ*. The measured values of the *T_g_* are listed in Table 7. The glass transition temperature slightly increased with the addition of the CNTs. Despite general agreement on the influence of the CNTs on most properties in rubber compounds, the effect of the CNTs on the glass transition temperature remains inconsistent. While some studies report no significant impact, others indicate an increase or decrease in the *T_g_* [13,49]. The increase in glass transition temperature can be attributed to reduced polymer mobility, enhanced crosslink density, and CNT–polymer interactions through van der Waals forces, π-π interactions, or hydrogen bonding, if functionalized CNTs with hydroxyl or carboxyl groups are used [13]. The thermal aging did not have a statistically significant influence on the value of the *T_g_*. The addition of the CNTs had the most significant impact on *tan δ* around the glass transition temperature, approximately between −65 °C and −30 °C, where an increase in CNT concentration led to a decrease in tan δ. Thermal aging also caused a slight decrease in *tan δ* within this temperature range. Below this range, nanocomposites exhibited higher *tan δ* values compared to NR-0, while above this range, no clear trend was observed.

### 3.6. Evaluation of the Surface Using Atomic Force Microscopy

**Figure 14:**
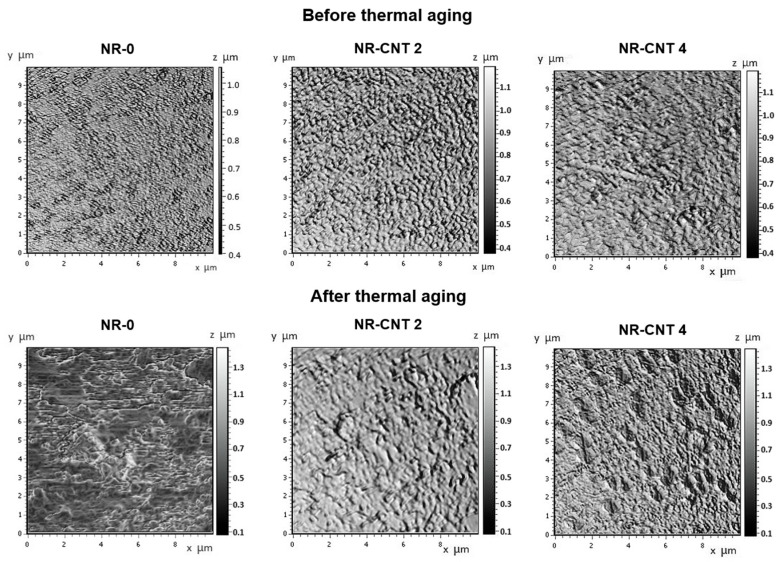
AFM images of surface before and after thermal aging.

The addition of the CNTs to rubber compounds can also affect their surface properties. The AFM was utilized to examine the topography of the compounds, both before and after 168 h of thermal aging at 100 °C. The AFM-obtained images are shown in Figure 14. The arithmetic mean deviation *Ra* and the root mean square deviation of the assessed profile *Rq* obtained by the AFM software (SurfaceXplorer 1.0.8.65) are presented in Table 8.

The *Ra* and *Rq* values of the NR-0 before thermal aging were 0.62 μm and 0.71 μm, respectively. The presence of CNTs led to the formation of slight microstructural irregularities and fine inhomogeneities, causing an increase in the roughness of the nanocomposites compared to the NR-0 compound. Specifically, the roughness of the NR-CNT 4 compound was *Ra* = 0.95 μm and *Rq* = 1.07 μm. After thermal aging, the NR-0 compound exhibited more pronounced changes than the compounds reinforced with the CNTs, both in terms of roughness values and visual inspection. The parameters *Ra* and *Rq* of the reference NR-0 compound increased by 66.13% and 57.75%, respectively, after thermal aging, while the *Ra* and *Rq* values of the NR-CNT 4 were only 9.47% and 7.47% higher after aging. The presence of the CNTs can improve the stability of the surface by reducing the degradation rate and preventing excessive roughness growth.

## 4. Conclusions

The results of this study demonstrate that prolonged exposure to elevated temperatures significantly affects the mechanical properties of rubber composites. The observed property changes indicate the degradation of the polymer matrix and a decline in the structural integrity of the rubber compound.

The addition of carbon nanotubes enhanced the mechanical properties of the rubber compound, particularly in terms of strength and resistance to deformation at standard operating temperatures. Even after thermal aging, the CNT-reinforced nanocomposites outperformed the reference compound without the CNTs. The rate of decline in tear strength and tensile strength due to aging was lower, and the CNTs also contributed to improved surface thermal stability, which is beneficial for sealing applications. These findings provide valuable insights for designing materials intended for long-term exposure to elevated temperatures.

Overall, this study confirms that temperature plays a critical role in determining the properties of rubber composites and that the CNTs can enhance their mechanical properties. These results can aid in the development of advanced materials for industrial applications where materials are exposed to temperature fluctuations. Future research should explore the development of more thermally stable polymer matrices, the impact of different stabilizers and antioxidants, and the functionalization of the CNTs to improve their compatibility with the polymer matrix. Additionally, investigating hybrid fillers, such as the CNTs combined with other nanomaterials, could further enhance mechanical and thermal properties. Finally, assessing the behavior of these nanocomposites under thermal cycling and combined environmental stresses, including humidity and UV radiation, will be beneficial for ensuring their reliability in real-world applications.

## Figures and Tables

**Table 1:** Composition of the compounds.

Component	Amount (phr)
Natural rubber (NR)	100
Carbon black filler (N 660)	30
Reclaimed rubber	5
Silica filler ULTRASIL	8
Resin	2
DAE—oil	5
Antiozonant—6PPD	1
Antioxidant—TMQ	1
Vulcanization activator—STEARIN	3
Vulcanizing agent—insoluble sulfur 67%	2
Sulfenamide vulcanization accelerator–CBS	1
Vulcanization activator—ZnO	3

**Table 2:** Ratios of carbon nanotubes (CNTs) in individual compounds.

Compound Name	NR-0	NR-CNT 1	NR-CNT 2	NR-CNT 3	NR-CNT 4
Ratio of CNTs (phr)	0	1	2	3	4

**Table 3:** Tensile test results before aging and after 168 h of aging at 100 °C.

Compound	NR-0	NR-CNT 1	NR-CNT 2	NR-CNT 3	NR-CNT 4
Tensile strength (MPa)					
Before aging	15.34 ± 0.41	15.85 ± 0.38	16.36 ± 0.31	16.85 ± 0.35	17.14 ± 0.39
After aging	13.65 ± 0.42	14.17 ± 0.37	14.82 ± 0.33	15.45 ± 0.32	16.12 ± 0.38
Elongation at break (%)					
Before aging	15.34 ± 0.41	15.85 ± 0.38	16.36 ± 0.31	16.85 ± 0.35	17.14 ± 0.39
After aging	13.65 ± 0.42	14.17 ± 0.37	14.82 ± 0.33	15.45 ± 0.32	16.12 ± 0.38
Tensile modulus (MPa)					
Before aging	15.34 ± 0.41	15.85 ± 0.38	16.36 ± 0.31	16.85 ± 0.35	17.14 ± 0.39
After aging	13.65 ± 0.42	14.17 ± 0.37	14.82 ± 0.33	15.45 ± 0.32	16.12 ± 0.38

**Table 4:** Hardness, tear strength, and rebound resilience test results before aging and after 168 h of aging at 100 °C.

Compound	NR-0	NR-CNT 1	NR-CNT 2	NR-CNT 3	NR-CNT 4
Hardness (Shore A)					
Before aging	50.21 ± 0.72	51.55 ± 0.83	52.96 ± 0.91	54.12 ± 0.89	55.78 ± 0.91
After aging	54.64 ± 0.85	55.38 ± 0.91	57.14 ± 0.94	58.78 ± 0.84	59.97 ± 0.89
Tear strength (kN/m)					
Before aging	64.54 ± 0.52	66.58 ± 0.59	70.23 ± 0.65	72.54 ± 0.61	73.99 ± 0.62
After aging	57.12 ± 0.57	61.25 ± 0.56	64.95 ± 0.61	67.25 ± 0.59	69.99 ± 0.58
Rebound resilience (%)					
Before aging	62.25 ± 0.87	60.95 ± 0.92	59.52 ± 0.74	58.25 ± 0.83	56.55 ± 0.78
After aging	58.75 ± 0.84	57.71 ± 0.85	56.01 ± 0.79	55.03 ± 0.90	53.02 ± 0.76

**Table 5:** Tensile set test results.

	NR-0	NR-CNT 1	NR-CNT 2	NR-CNT 3	NR-CNT 4
Tensile set after 25% elongation (%)					
23 °C	11.44 ± 0.12	11.12 ± 0.11	10.95 ± 0.13	10.52 ± 0.11	10.28 ± 0.13
70 °C	12.32 ± 0.15	12.02 ± 0.11	11.59 ± 0.13	11.02 ± 0.14	10.89 ± 0.13
100 °C	25.21 ± 0.16	24.52 ± 0.13	23.02 ± 0.14	22.89 ± 0.16	22.23 ± 0.15
Tensile set after 50% elongation (%)					
23 °C	16.21 ± 0.15	15.82 ± 0.13	15.25 ± 0. 16	14.98 ± 0.13	14.55 ± 0.15
70 °C	18.02 ± 0.13	17.55 ± 0.15	17.01 ± 0.12	16.65 ± 0.13	15.88 ± 0.16
100 °C	34.12 ± 0.17	33.85 ± 0.16	32.75 ± 0.13	31.54 ± 0. 18	30.11 ± 0.15

**Table 6:** Compression set test results.

	NR-0	NR-CNT 1	NR-CNT 2	NR-CNT 3	NR-CNT 4
Compression set after 25% compression strain (%)					
23 °C	12.58 ± 0.14	12.05 ± 0.15	11.23 ± 0.14	11.02 ± 0.16	10.99 ± 0.15
70 °C	14.05 ± 0.16	13.84 ± 0.15	12.78 ± 0.15	12.03 ± 0.16	11.95 ± 0.15
100 °C	28.32 ± 0.16	27.52 ± 0.17	26.45 ± 0.15	25.87 ± 0.18	24.12 ± 0.17

**Table 7:** Values of glass transition temperature *T_g_*.

Compound	NR-0	NR-CNT 1	NR-CNT 2	NR-CNT 3	NR-CNT 4
*T_g_* before aging	−57.1 ± 1.2 °C	−54.8 ± 0.9 °C	−53.1 ± 1.2 °C	−51.6 ± 1.3 °C	−50.3 ± 1.1 °C
*T_g_* after aging	−55.4 ± 1.5 °C	−52.7 ± 1.1 °C	−50.2 ± 1.0 °C	−49.7 ± 1.4 °C	−48.3 ± 0.9 °C

**Table 8:** Roughness parameters obtained by the AFM.

Compound	*Ra* (μm)		*Rq* (μm)	
	Before Aging	After Aging	Before Aging	After Aging
NR-0	0.62 ± 0.01	1.03 ± 0.05	0.71 ± 0.03	1.12 ± 0.06
NR-CNT 1	0.69 ± 0.02	0.88 ± 0.04	0.78 ± 0.02	0.96 ± 0.03
NR-CNT 2	0.76 ± 0.03	0.95 ± 0.04	0.84 ± 0.03	1.04 ± 0.04
NR-CNT 3	0.84 ± 0.02	0.99 ± 0.03	0.95 ± 0.04	1.09 ± 0.05
NR-CNT 4	0.95 ± 0.02	1.04 ± 0.02	1.07 ± 0.03	1.15 ± 0.03

## Data Availability

Data are contained within the article.
